# Formative Evaluation of Clinician Experience with Integrating Family History-Based Clinical Decision Support into Clinical Practice

**DOI:** 10.3390/jpm4020115

**Published:** 2014-03-26

**Authors:** Megan Doerr, Emily Edelman, Emily Gabitzsch, Charis Eng, Kathryn Teng

**Affiliations:** 1 Center for Personalized Healthcare, Medicine Institute, Cleveland Clinic, 9500 Euclid Ave., Cleveland, OH 44195, USA; E-Mails: gabitze@ccf.org (E.G.); tengk@ccf.org (K.T.); 2 Genomic Medicine Institute, Cleveland Clinic, 9500 Euclid Ave., Cleveland, OH 44195, USA; E-Mail: engc@ccf.org; 3 Genomics Education, the Jackson Laboratory, 600 Main Street, Bar Harbor, ME 04609-1500, USA; E-Mail: emily.edelman@jax.org; 4 National Coalition for Health Professional Education in Genetics, 2360 W. Joppa Road, Suite 320, Lutherville, MD 21093, USA

**Keywords:** family health history, clinical decision support, implementation, risk stratification, barriers, facilitators

## Abstract

Family health history is a leading predictor of disease risk. Nonetheless, it is underutilized to guide care and, therefore, is ripe for health information technology intervention. To fill the family health history practice gap, Cleveland Clinic has developed a family health history collection and clinical decision support tool, *MyFamily*. This report describes the impact and process of implementing *MyFamily* into primary care, cancer survivorship and cancer genetics clinics. Ten providers participated in semi-structured interviews that were analyzed to identify opportunities for process improvement. Participants universally noted positive effects on patient care, including increases in quality, personalization of care and patient engagement. The impact on clinical workflow varied by practice setting, with differences observed in the ease of integration and the use of specific report elements. Tension between the length of the report and desired detail was appreciated. Barriers and facilitators to the process of implementation were noted, dominated by the theme of increased integration with the electronic medical record. These results fed real-time improvement cycles to reinforce clinician use. This model will be applied in future institutional efforts to integrate clinical genomic applications into practice and may be useful for other institutions considering the implementation of tools for personalizing medical management.

## 1. Introduction

There is increasing acceptance among practicing clinicians that health informatics, in general, and clinical decision support (CDS) tools, in particular, can be used to improve the quality and consistency of healthcare delivery, playing a fundamental role in both standardizing and personalizing approaches to care [1,2,3,4,5,6]. Despite its acknowledged importance for identifying risk factors for eight of the top ten leading causes of death in the U.S., family health history is unsystematically collected and inconsistently applied to guide medical care [7,8,9,10]. For this reason, family health history is ripe for health informatics-based interventions, including CDS [11,12,13,14]. Examples include the CDC’s *Family Healthware*^™^, Duke’s *MeTree*, Intermountain Healthcare’s *OurFamilyTree* and *The Pregnancy and Health Profile* developed by the March of Dimes, the National Coalition for Health Professional Education in Genetics, Partners Healthcare and Genetic Alliance. All of these tools are electronic, patient-entered family health history questionnaires. All are principally or in part focused on informing preventive care planning for adult patients in primary care. All but *OurFamilyTree* include CDS, although none provide this support directly through the electronic medical record (EMR).

*MyFamily* is a family health history collection tool that delivers CDS through the EMR at the point of care developed by Cleveland Clinic (CC) with the aim of standardizing the collection and use of family history to guide care planning. *MyFamily* was opened for clinical use in adult care at CC in fall, 2012.

### 1.1. Description of Application

Patients are invited to complete the web-based *MyFamily* questionnaire via the CC patient portal up to two weeks in advance of a scheduled appointment with a participating clinician; only patients aged 18 and older qualify for invitation. Patients answer questions about general health parameters and then are led through constructing their family tree. Finally, patients are asked family health history questions specific to the upcoming appointment. There is a “save and come back” feature to facilitate patient data collection. All patient entered data is saved and represented in subsequent logins and for succeeding *MyFamily* invitations.

**Figure 1:**
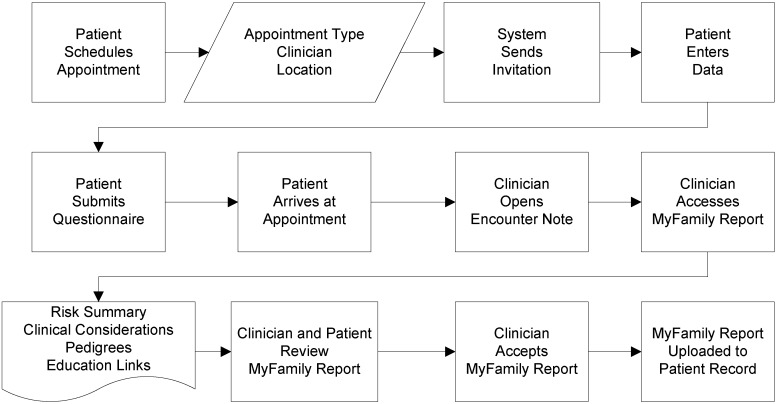
*MyFamily* workflow schema showing clinician acceptance of *MyFamily* report.

For those patients who submit their questionnaires, the clinician receives a custom, patient-specific report through the EMR, accessed through the encounter note. The *MyFamily* report includes: (1) a dynamically ranked risk snapshot listing the patient’s risk for each condition assessed; (2) clinical considerations, rationale and potentially applicable diagnosis code(s); (3) family health history in both pedigree and list formats; and (4) additional educational content about each condition for both clinicians and patients. Following review and use in the clinical encounter, the clinician can accept the documents, thereby uploading them permanently into the EMR (Figure 1), request review from the *MyFamily* staff prior to acceptance or decline the report, preventing it from becoming part of the patient’s EMR. Screen shots of the application are shown in Appendix A.

The clinical content included in the *MyFamily* report, e.g., risk assessment, clinical considerations and rationale, were developed by teams of CC clinicians, which included one or more subject matter experts, one or more primary care clinicians and genetic counselors. For example, for the hereditary nonpolyposis colorectal cancer syndrome content set, this was developed by a gastroenterologist and genetic counselors with a special interest in hereditary colorectal cancer syndromes and a primary care physician. Using standardized templates informed by the Institute of Medicine’s Clinical Practice Guidelines We Can Trust, each team assessed existing clinical guidelines and primary literature and developed risk assessment and CDS content sets consistent with CC’s clinical practice patterns [15]. CC’s Clinical Practice Committee further reviewed select clinical content sets for conditions where wide variation in clinical practice had been observed within the institution.

The conditions included in *MyFamily* were selected by the workgroup of practicing primary care clinicians and the *MyFamily* clinical development staff. The mandate of the group was to select conditions that would inform adult preventive care planning in the well-adult setting using family health history. The group returned more than 30 potential conditions; eight were developed before the initial deployment (Table 1), with 4–6 additional conditions added dynamically each year.

**Table 1:** Conditions assessed by *MyFamily* at the start of implementation.

Familial breast cancer
Familial colorectal cancer
Familial endometrial cancer
Familial ovarian cancer
Hereditary breast and ovarian cancer syndrome
Hereditary nonpolyposis colorectal cancer syndrome
Abdominal aortic aneurysm *
Diabetes *

* included in primary care (PC) implementation only.

### 1.2. Aims of the Study

The implementation of *MyFamily* was accompanied by a formative evaluation assessing clinician usability, including the impact on clinic flow and perceptions of usefulness and value. The ultimate goal of the study was to identify barriers and facilitators to maximizing clinical adoption to feed iterative improvement cycles, consistent with the Consolidated Framework for Implementation Research (CFIR) proposed by Damschroder and colleagues [16].

## 2. Methods

### 2.1. Design

The formative evaluation employed a novel mixed methods approach, including both quantitative and qualitative measures. Based on clinician needs and preferences, direct observation, interview and quantitative metrics were selected as the methods of assessment. The CFIR advocates for formative evaluations as a way to assess the effectiveness of a given intervention within and across settings and iteratively optimize the intervention to each setting. The focus of this evaluation was to identify the “adaptable periphery” as quickly as possible to thereby expedite the adaptive change of the *MyFamily* application, with the ultimate goal of prolonging sustainability and promoting dissemination of the application across CC. As this was a quality improvement project, clinician participants acknowledged involvement prior to each interview and were re-contacted to confirm intent to participate during the preparation of this manuscript. Data collection from patients underwent CC Institutional Review Board review and approval (07-591). This manuscript reports on the qualitative and quantitative findings from semi-structured interviews with clinicians 30–45 days following their first use of *MyFamily* in the clinic.

### 2.2. Participant Selection

Three provider groups were selected for the *MyFamily* pilot, all of which focus on well-adult preventive care planning: primary care clinicians, cancer genetic counselors and cancer survivorship clinicians—CC’s formal handoff of patients from primary management within oncology back to the primary care setting. The purpose of selecting three diverse settings within our trialability test was to describe the broad space of implementation of *MyFamily* within CC and the specific needs of clinicians in these settings *vis-à-vis* the application. Each setting is complex in its own way. Within primary care at CC, the “medical home” model of care increases the need for adaptation of the tool to team care, as well as meeting the demands of a clinic where family history assessment is one small part of the total patient visit. Cancer genetic counselors, by contrast, do not have a team care model of practice at CC and, consistent with professional society standards, have a primary focus on family health history gathering and assessment within their encounters. The cancer survivorship clinic was included as a potential “mid-point” between these two groups. At CC, this clinic is staffed by advanced practice nurses with an interest in hereditary cancer, with the oversight of CC oncologists; hence, neither a team care nor sole provider model. Within the survivorship visit itself, there are several proscribed areas for assessment, consistent with national standards, which include a specific focus on family history gathering and risk assessment. Due to this, it was hypothesized that the *MyFamily* clinical needs of cancer survivorship providers would be more like those of genetic counselors than of primary care clinicians. A total of 10 practicing clinicians from primary care (PC), genetic counseling (GC) and cancer survivorship (CS) participated in the formative evaluation.

### 2.3. Interview Procedures

All participants were interviewed in a one-on-one setting by one of two researchers (Megan Doerr and Emily Gabitzsch) Most participants (*n* = 9) were interviewed in person, with one participant interviewed by phone. The interviewers followed a semi-structured interview guide covering five domains of clinical experience. Each interview section contained both closed- and open-ended questions, with a maximum of 38 structured questions included. Due to the time constraints of practicing clinicians, the interview was designed to be completed within 30 min. The interview guide includes 20 closed-response questions employing Likert-scale and or other fixed response sets, 8 of which have potential prompts, and 18 open-response questions. Closed-response questions included focused drill down prompts on specific themes germane to the implementation study, e.g., specific adaptive needs from the application to the practice setting, barriers and facilitators to implementation (Appendix B). The results regarding ease of use, perceived usefulness and value and impact on clinic flow are reported in this manuscript and include both the quantitative and qualitative data gathered over the course of the interviews.

### 2.4. Data Analysis

Interviews ranged in length from 12.5 to 33.5 min (mean: 24.9 min; median: 24.7 min) and were conducted at the clinician’s primary or secondary worksite between December, 2012, and April, 2013. All interviews were recorded, transcribed, and de-identified using interview identification numbers. The first author (Megan Doerr) managed the audio recordings, their transcription and kept detailed notes about the interviews themselves. Audio and transcriptions were compared by the data analysis team (Megan Doerr and Emily Edelman) and discrepancies resolved by consensus.

The data analysis team read transcripts independently, noting primary and secondary codes, findings and memos. They then met and discussed their analyses, identifying concordant/discordant coding. Together, they refined primary and secondary codes and findings. Each member of the data analysis team categorized findings, primary and secondary codes into broader themes and again cross compared. After reaching consensus on the number and spectrum of refined themes, the data analysis team again examined transcripts to ensure thematic consistency. No new themes emerged, and it was determined that the analysis was complete with inclusive, robust themes identified. The data analysis team assessed themes from the population of 10 as a whole, as well as within each specialty area.

## 3. Results

### 3.1. Sample Characteristics

A total of 10 clinicians participated in the formative evaluation, including four primary care physicians, four genetic counselors and two advanced practice nurses. These clinicians serve primary care (family medicine and internal medicine; four physicians and one advanced practice nurse), cancer genetics (four genetic counselors) and cancer survivorship (one advanced practice nurse) clinics across CC at both the main hospital and regional family health centers. Clinicians had between 1 and 30+ years of clinical experience; all clinicians had been practicing in their current clinical setting for at least six months at the time of interview.

### 3.2. Summary of Findings

Clinician themes addressed three major outcomes of implementation: the impact of *MyFamily* on patient care; the impact of *MyFamily* on the clinical encounter and workflow; and the general process of implementation. Major themes and findings are summarized below (Table 2).

**Table 2:** Overview of themes and findings.

Themes	Impact on patient care	Impact on clinical encounter and clinician workflow	Process of implementation
*Findings*	Quality Patient engagement Institutional impact	Impact on workflow Time Navigation Length and detail Risk assessment Pedigrees	Barriers Facilitators Future functions

#### 3.2.1. Impact on Patient Care

##### 3.2.1.1. Quality

*MyFamily* was seen as facilitating an increase in the quality of care by all those interviewed. Three distinct domains of improved quality were commented upon by both PC and GC clinicians. Both groups of clinicians felt that *MyFamily*: (1) ensured the provision of standard-of-care and equal care to all patients; (2) impacted the accuracy of family health history reported by the patient; and (3) enabled the provision of cross-disciplinary care (Table 3).

**Table 3:** Primary care provider (PC) and cancer genetic counselor (GC) data addressing quality of care.

Title	PC	GC
Provides standard and equal care to patients	“Often times that [family health history conversation] doesn’t happen especially if patients have a lot of medical problems, you know we might not spend as much time on preventative care… having that opportunity to sit down with them and their family history kinda gives that opportunity to have that discussion.” (PC101)	“I can see it helping a lot of people that… would never come to attention if they are healthy individuals with concerning family history… something like [*MyFamily*] would pick [these patients] up.” (GC107) “… [*MyFamily* is] a very good educational opportunity for providers who did not previously really understand who needed to be referred to genetics.” (GC108)
Accuracy of family health history	“…if that patient is starting that dialogue at home where they have access to the information, talking to families, friends, relatives, they could possibly do it more accurately.” (PC102)	“…it increases my confidence that we are getting accurate information because I think it forces patients to think about it ahead of time, and they ask family members for information and they come in better prepared to answer our questions, so in that sense, I think [*MyFamily*] improves [quality].” (GC108)
Supports cross-disciplinary care	“…having a more detailed history and having that analyzed and then given to me… helps me decide… should I send him off to a specialist sooner than later or should I really be on their case to get that colonoscopy done and that sort of stuff.” (PC104)	“…I think [the use of *MyFamily*] is really going to improve our referrals in terms of quantity and quality.” (GC108) “A patient “was referred to me from their primary care physician after completing [*MyFamily*]. [I reviewed the patient’s *MyFamily* report] because I was trying to get a better understanding of why that patient was referred to genetic counseling.” (GC105)

Regarding the provision of standard-of-care and equal care, PCs commented on *MyFamily* facilitating a useful, consistent, targeted family health history discussion for every patient (Table 3). Similarly, GCs spoke of the potential for *MyFamily* to detect healthy individuals with a concerning family history previously unrecognized as having a clinically significant disease risk and to identify these individuals as appropriate to refer to genetics for further evaluation. GCs predicted that systematic use of *MyFamily* would improve the quality of referrals by educating non-genetics clinicians about family history risk assessment. The CS interview revealed similar themes, with this clinician supporting *MyFamily*’s impact on the consistent delivery of standard-of-care, stating:

“The people who take the time [to complete *MyFamily*], it’s going to treat them equally ‘cause everybody is going to be looked at as a blank slate, no names, no numbers, none of that attached to that.”

(CS110)

Both PCs and GCs addressed the potential for *MyFamily* to improve the accuracy of family health history information documented in the medical record (Table 3). They pointed to patients having time to talk with relatives while completing the tool as the key to improving the accuracy of the patient report overall. On the topic of the accuracy of family health history reporting by patients, the CS brought a different perspective, commenting on the risk of patient self-censoring to speed the completion of the questionnaire and the potential impact of those omissions on the accuracy of risk assessment returned by the application,

“I would fear that if it does become [too] time consuming, that [patients] are going to be like, ‘You know what, I’m just going to put down the people who have cancer and the other people don’t really matter.’ Although the other people do matter…”

(CS110)

In the provision of cross-disciplinary care, PCs highlighted *MyFamily*’s risk assessment and CDS facilitating their own decision making about referral for specialist care (Table 3). GCs saw *MyFamily* as improving the quality and quantity of referrals they received, as well as acting as an information bridge between primary care and the specialist setting. The CS supported both of these views, stating:

“[*MyFamily*] definitely increases the quality of care. Again, I think that it brings out the fact that we are multi-disciplinary and that we are interested in the whole [patient]; every piece that we can bring to a patient we are bringing to them.”

(CS110)

These narratives are supported by quantitative data from the clinician interviews. When asked, “On a scale of 1 (very helpful) to 5 (not helpful at all), how helpful is the RR [risk reference] in identifying patients appropriate for referral to subspecialties and other services?” the PC average response was 1.8 (range: 1–3); the GC average response was 2.0 (range: 2); and CS was 1.

##### 3.2.1.2. Patient Engagement

All clinicians interviewed commented on *MyFamily* as a potential method of increasing patient engagement, as shown by the examples in Table 4.

**Table 4:** Patient engagement.

PC	GC
“I think that it’s a great way to really engage patients and to really help them to see [that] what they are doing, the work that they are doing pre-visit, is really helping us to make this preventative [care] plan with them. So, I really like that aspect of it…. I think they enjoyed seeing their results of their efforts during the clinical encounter, I think they really appreciated that.” (PC101)	“[my favorite part is] the fact that the patients are more engaged…. They have been more active in their health care. They have been more active in their family history gathering. They come in having a better idea of what to expect.” (GC107)

One PC emphasized the paramount importance of patient engagement to CC and the national healthcare system as a whole:

“I find [*MyFamily*] incredibly useful because it engages the patient in their health which ultimately [supports] the value based operations that the Cleveland Clinic is moving towards. If we don’t partner with our patients, their employers, our local governments, our communities, our churches, our neighbors, we will not be able to afford health care in this country…”

(PC102)

The CS was one of a handful of providers who also commented that sustaining patient engagement depends both on the patient and on the clinician’s reaction to that engagement within the clinical encounter:

“…if the patients are taking the effort… to make sure that the clinicians who are getting the information are discussing it. Because otherwise the patient will stop filling these things out, if they fill it out and they have to ask [their clinician] about it or it never even gets brought up [by the clinician].”

(CS110)

##### 3.2.1.3. Institutional Impact

PC and CS clinician groups connected the *MyFamily* initiative to the institutional goals of CC, with the application described as reinforcing and supporting clinicians in meeting targets in clinical efficiency and the personalization of care.

One of the consistent areas of comment from PCs regarding *MyFamily* was its role as a tool to assist with the institution’s drive to increase clinical efficiency.

“…if we can do [family history risk assessment] in a uniform way and [patients] can understand that if someone has reviewed this, this is constantly being updated, I think it will save a lot of time for the system.”

(PC101)

The CS agreed,

“…it allows me to tell the patient that [his/her family history has] already been reviewed and this is what it is. It’s not a gray area [where I would say] I will get back to you, let me review this with a genetic counselor—the decision has already been made. … I think it saves time because there is not the checking, double checking, calling the patient back.”

(CS110)

PCs were asked specifically about *MyFamily’s* effectiveness in helping meet “health maintenance requirements,” CC’s electronic system for preventive care management; PCs are responsible for fulfilling this reporting function within CC. Health maintenance targets are publicized across primary care, and clinicians discuss their individual efforts toward achieving these goals as part of the annual review process. When asked to rate *MyFamily*’s helpfulness in meeting health maintenance requirements on a scale of 1 (very helpful) to 5 (not very helpful at all), PC average response was 2.0 (range 1–3). Several PCs highlighted that continuing to enhance electronic integration to specific points within the health maintenance system would increase the value gained from the application’s use in clinical practice.

*MyFamily*’s effectiveness in the personalization of care was another institutional goal commented on by both PC and CS clinicians:

“…having a more detailed history and having that analyzed and then given to me… it personalizes their care which is obviously the whole idea behind it.”

(PC104)

Institutional impact statements were limited to PC and CS clinicians, perhaps due to the higher perceived usefulness of the tool to this group as compared with the GCs. When asked to assess the usefulness of the application to their practice from 1 (very useful) to 5 (not very useful at all), the PC response average was 1.8 (range 1–3), while the GC response average was 3.6 (range 2.5–5). The GCs’s perception of the application’s lack of usefulness is further described in the next section, specifically within the review of data relating to *MyFamily* generated pedigrees.

#### 3.2.2. Impact on Clinical Encounter and Clinician Workflow

##### 3.2.2.1. Impact on Workflow

Clinicians were asked to assess and describe the impact of *MyFamily* on their clinical workflow (Table 5). No clinician noted that *MyFamily* introduced novel topics to their clinical encounters. PC and CS clinicians reported natural integration of the application into their existing clinical workflows, fitting *MyFamily* report review in during their regularly scheduled discussion of family health history. GCs and the CS needed to spend additional time reviewing and updating *MyFamily* before or after to the clinical encounter in order to have an efficient encounter with the patient; with this additional effort outside of the encounter, the encounter itself was not impacted.

##### 3.2.2.2. Time

Clinicians had varying perspectives on the time needed to review *MyFamily* within the clinical encounter (Table 5). The clinician (PC) who had had the greatest number of patients using the application during the study period felt that it saved time within the visit. PCs felt use was becoming increasingly efficient over time. In close-ended questioning, four clinicians (1 PC; 3 GCs) were neutral on time spent; three (PCs) noted an extension in time spent talking about family history, without an extension in overall encounter time; and one (GC) reported an increase in time spent. Of those PCs noting an extension in discussion time, all described a benefit to the encounter. The CS clinician reported using *MyFamily* saves time in patient care, as described above in the clinical efficiency benefits of the system.

##### 3.2.2.3. Navigation

Although navigation was not a common theme among GCs, as described above, PC participants recounted an initial learning period followed by increasingly facile navigation of *MyFamily*:

“But once you click the *MyFamily*, open it up and really see how everything is kind of bulleted, where the risk reference is, I think after that, it gets a little easy. So when I first opened it, I did not know what to look for and look at… Like I said, when you first navigate through it, it's kind of confusing on what we’re looking at… I think that’s the initial thing, just really knowing what we’re looking at and how to navigate through it.”

(PC103)

Length was the primary hurdle to successful navigation and use:

“…until I became more accustomed to it, initially I was taking back a little bit by the length of the document. Once I became more familiar with it and was able to navigate it and know where to look for the information that was most pertinent, it became much more user friendly.”

(PC102)

**Table 5:** Primary care provider (PC) and cancer genetic counselor (GC) data addressing workflow and usability. EMR, electronic medical record.

Title	PC	GC
Impact on Workflow and Time	“…part of my routine when I do a physical, is review family history, so I look at my family history record in [the EMR] and then I go to [the *MyFamily* report] and see if there is anything else to add.” (PC109) “But it’s time well spent. It’s time that is spent reviewing the family history and talking to patients about their disease risk.” (PC101)	“…the actual interaction with the patient has not changed much…[but afterwards] what I have been doing is providing our assistant with [the pedigree] to have her re-enter it…[to] generate a new pedigree, and that process takes a while because she is busy. So, the flow has not been as quick as I would typically like.” (GC105) “…if there were multiple pedigrees for different conditions, I would try to consolidate them onto one before I saw the patient. Other times, I would have it printed out and with the patient, we would go through each one and consolidate them… I have had to…re-draw the whole thing based on that information that we collected...” (GC108)
Risk Assessment	“I really have been looking for something that can help with clinical decision making… I think [the risk assessment] is a valuable addition to our armamentarium of… taking care of the patient.” (PC104)	“…but for the risk assessment… that is my job… I think [the risk assessment] is much more applicable or much more helpful, I guess, to, like, a generalist.” (GC107) “Yeah, if it is too confusing on the risk reference, I will just not accept it because if it is confusing to me, I figure it is going to be confusing for other people especially if it is [contradicting my own risk assessment].” (GC106)
Pedigrees	“…it is much easier for me to look at a pedigree and have a clearer picture of why a person was flagged as high risk… which surprises me, because I don’t normally use pedigrees, but I have found it to be very useful information.” (PC101)	“I picked the [pedigree] that has the most information on it. I print it out, and then I look at the other pedigrees and handwrite in the information from the other pedigrees, and then I used that as my structure to go over with the patient.” (GC105) “…but I think the layout is not effective and not useful because you end up redrawing it and it does not really save too much time during the appointment. You could probably draw it yourself by asking questions just as fast.” (GC106)

##### 3.2.2.4. Length and Detail

Six participants (2 PC, 3 GC, 1 CS) described the length of the *MyFamily* report as “too long,” three (3 PC) as “okay” and one (GC) as “appropriate.” Clinicians found the report easy to understand, demonstrated by a 1.80 understandability score for both PCs (range: 1–3) and GCs (range: 1–3) on a Likert scale of 1 (very easy to understand) to 5 (very difficult to understand). At the same time, several clinicians noted desiring additional detail about how risk scores were generated and/or access to the granular level detail inputted by patients. Clinicians 108 and 101 illustrate this tension between length and detail:

Length: “I think it is too long. I think…that is not reasonable to expect a [clinician] who sees a ton of patients every day to get through easily. I mean though there is a summary page and I think that is very helpful, but I think there is also really good information on the individual pages that, you know, they have to, you know, scroll through…*”*

(GC108)

Detail: “There are certain parts, pieces of information that the patient says they inputted but does not pull through in to my screen when I open the *MyFamily* document… It seems like the output to me is not equivalent to what the patient inputs.”

(GC108)

Length: “…I find that I just, I look at the scores and I pick maybe the top two and probably ignore the rest.”

(PC101)

Detail: “…there is a section that says, ‘why is your person flagged’, but I don’t find that there is enough granular information in there to really counsel the patient about why they were at an increased risk.”

(PC101)

##### 3.2.2.5. Risk Assessment

In quantitative questioning, the average agreement score with *MyFamily’s* clinical risk assessment and recommendations for PCs was 1.6 (range: 1–2) and 2.1 (1.5–2.5) for GCs on a Likert scale of 1 (strongly agree) to 5 (strongly disagree). PC and CS clinicians (Table 5) described the risk assessment component as one input of many into their decision-making. One PC even considered the way in which risk assessment could be used to extend the time to screen for patients for those at lowest risk.

Clinician 109:

“My hope [is that] we could get to the point where we have an individual plan for patients that would change the way we do screening. I would like to see it to the point where well, you know, this 50-year old woman maybe really doesn’t need a colonoscopy, maybe we could wait until 55 for this person.”

Interviewer:

“So you would really be able to stratify high-risk populations from general risk populations and change your screening to meet those two populations?”

Clinician 109:

“Yes.”

Most genetic counselors saw the risk assessment provided in the *MyFamily* report as, at best, redundant to their clinical risk assessment and, at worst, contradictory to their risk assessment (Table 5). Most GCs did not use the *MyFamily* report other than to extract the family history data from the pedigree.

##### 3.2.2.6. Pedigrees

In the initial *MyFamily* deployment, the pedigree drawing software improperly rendered some essential pedigree formatting elements. Additionally, separate pedigrees of each of the conditions assessed were presented in the *MyFamily* report. As GC and CS clinicians universally described pedigrees as critical tools for clinical care, these two facts created a barrier to successful implementation within this group, with the pre- or post-visit time needed to re-render pedigrees being a primary hurdle (Table 5). Not only were the separate pedigrees a barrier to effective clinical use, one clinician felt they hampered clinician appreciation of the complete patient presentation:

“…with the pedigree being so divided up that it really kinda compartmentalizes the patient’s care… Where if you have a pedigree that’s all on the same page, you can really get a grasp of what that patient is dealing with…if you are looking helping the patient, to look at only one problem in their health history, I’m not really sure it’s doing them a whole lot of good.”

(CS110)

These challenges opened the door to non-compliance, where GCs are averse to using the pedigree in the encounter, as illustrated by these quotes:

“…if I left it as all the separate pedigrees, I would not use it.”

(GC107)

“…if the pedigree is good, I would accept [the MyFamily report]. If not, I would just not use [MyFamily for that patient].”

(GC106)

Despite these challenges, GC and CS clinicians noted a benefit from having family health history and family structure data about patients in advance of their appointment:

“I do like the ability to look at things before the patient checks in… because I have had an opportunity to review it before they have arrived, it doesn’t interrupt at all. I am prepared to go in and have that discussion.”

(CS110)

If mentioned at all, PC clinicians described pedigrees as learning/understanding tools, but were not affected by the pedigree rendering challenges (Table 5).

#### 3.2.3. Process of Implementation

##### 3.2.3.1. Barriers

Few clinicians explicitly identified barriers to wider implementation. The issues that were articulated by participants fell primarily at the system level, focusing on EMR integration and standardization. Clinicians also expressed that lack of clinician engagement would be a barrier to the success of implementation:

“…particularly in regards to primary care, patients are taking the time to fill this out, but if the physician is too busy to look it over or review it, this might not be a physician that [MyFamily] should be sent out for…”

(CS110)

##### 3.2.3.2. Facilitators

All clinicians identified the *MyFamily* staff as an important facilitator of implementation. The training team’s one-on-one face time with clinicians, their physical presence in clinic and flexible schedules to meet clinic needs, as well as the training team’s direct patient support were all called out as features of successful implementation. As one participant stated,

“I have had a lot of one-on-one TLC from you guys.”

(CS110)

##### 3.2.3.3. Future Functions

Clinicians provided detailed feedback about desired technical enhancements, with PC and CS requests centering on increasing the number of EMR integration points, including automatic health maintenance updating and “clickable” order sets. All groups requested existing external web-based risk assessment tools (e.g., National Heart, Lung, and Blood Institute/Framingham Risk Score) to be automatically run and the results integrated into the *MyFamily* report. GC and CS clinicians demanded consistently accurate pedigree rendering and a single, integrated pedigree showing all conditions. 

Future function suggestions were actively collected, analyzed and processed during the implementation study period. Application improvements identified as critical to clinician acceptance and use were released in real time. This updating of the system is illustrated by the shift in feedback regarding the pedigree; one GC was interviewed following the resolution of pedigree malformatting and the institution of a “consolidated” pedigree showing all conditions for the patient:

“I have a better jumping off point and at least the family structure basically down. So it, I think, especially with the newly improved pedigrees, [using *MyFamily* for patient care] will be faster.”

(GC108)

## 4. Discussion

We studied the implementation of *MyFamily*, a family health history collection and CDS tool, using qualitative and quantitative data describing clinician experience, usability and utility. This study demonstrates that clinicians, especially PC, view the system as highly usable, fitting naturally into their existing workflows and personal practice patterns.

Clinicians universally agreed that *MyFamily* would increase the quality and consistency of care received by patients, appropriately flagging those at high risk for additional screening/referral, more in-depth/targeted counseling, facilitating a personalized approach to risk management and allowing for appropriate reassurance of the “worried well.” PC and CS clinicians commented on the shift in their family health history conversations from gathering to using the information provided to make management decisions, as well as having more nuanced conversations about the risks pertinent to their particular patient. These findings are consistent with the reported outcomes of increased quality and access described in the implementation of similar family health history CDS tools into clinical practice [11,17,18,19]. One clinician (PC109) projected benefits of individualization care even further, predicting that eventually the data gathered from the system could be used to reduce interventions below current population guidelines for the lowest risk patients. This finding was unique to this participant, but is an intriguing point worthy of further consideration given the shifting landscape of healthcare in the United States. The potential for reducing intervention below current population guidelines could be a significant cost-savings for healthcare generally and have a positive impact on those lowest-risk patients with commensurate reduction in false positives and unnecessary diagnostic follow-up.

One important theme of this implementation was the tension between the length and detail of the information provided in the *MyFamily* report. The majority of clinicians felt the *MyFamily* report was too long and yet simultaneously expressed a desire for additional detail, including several requests for the inclusion of all of the individual patient inputs. This theme was particularly notable, as some of the GC clinicians who took part in the pilot were members of the risk assessment algorithm development teams and, as such, would have had intimate knowledge of the clinical criteria applied and risk assessment run to determine any given risk assessment result. In their widely cited systematic review identifying CDS features associated with improving patient care, Kawamoto and colleagues described two communication content features, “justification of decision support via provision of reasoning” and “justification of decision support via provision of research evidence” which might be descriptive of this self-contradictory wish for a brief report, as well as voluminous detail [20]. In their analysis, neither of these two features were identified as critical to CDS success, but as the essential feature set (decision support provided automatically as part of the clinician workflow, decision support delivered at the time and location of decision-making, actionable recommendations provided and computer based) becomes the standard design requirement of all CDS tools, communication content features may become important facilitators or barriers to successful implementation. Certainly at the time of their 2005 review, only 7% of the 88 unique interventions studied included “justification of decision support via provision of research evidence” with 100% of those CDS that included this feature having successful implementation. 

One possible future strategy to facilitate clinicians’ desired access to detail while managing their perception of report length is to explore advanced information technology solutions supporting a truly layered and branched approach to the presentation of CDS information. This approach is supported by McDermott and colleagues’ recent investigation of a computer delivered intervention for guideline implementation in general practice [21]. The team presented a multi-layered “prompt” system to encourage the use of practice guidelines in which clinicians were able to self-direct access from summary-level digests to various granular-level supporting information. The authors conclude that clinician preference for this system is consistent with the social cognitive theory “control of environment,” noting this as an important indicator of successful behavior change. 

Nonetheless, it may not be possible to completely eliminate this clinician-based tension between the need for concise action-focused messages and the desire to oversee and verify each detail underlying every risk assessment. The essential mandate of CDS is to give clinicians actionable solutions to save time and standardize practice; if clinicians feel compelled to “own” all the detail behind the risk assessment, this may undermine the benefit of the CDS to the healthcare system it is attempting to streamline and standardize.

The implementation team took an active approach to enacting real-time enhancements to retain, boost and reinforce clinician engagement in the *MyFamily* application during the implementation study period. This continuous cycle of improvement is illustrated by the challenges described at the beginning of the implementation period of malformatted pedigrees and their condition-specific presentation, undermining non-PC engagement and use of *MyFamily*, to the increasing acceptance and engagement seen in those clinicians interviewed following the enhancement launch. Approaching implementation as a dynamic cycle of engagement and enhancement between clinicians, clinical implementation team members and information technologists was critical to the success of the implementation, consistent with the predictions of Damschroder and colleagues based on their Consolidated Framework for Implementation Research [16].

One of the strengths and limitations of this study is the diversity of provider types who participated in the implementation. It was beneficial to have perspectives from three different disciplines, but because of the small sample size, data saturation is a limitation of this study. In planned summative evaluations, we will solicit a greater number of providers from each discipline to address this concern. A second limitation is that the pilot group included several members of the risk assessment algorithm development team. Because of their involvement in the design of the tool, these clinicians may have been more positively inclined to the intervention. As the development and implementation took place within a single institution, this may limit the generalizability of this study’s findings. Another challenge with the design of this study is the limited time availability of clinician participants, especially primary care providers, to participate in interviews. While our design aimed to limit interviews to approximately 30 min or less to meet the practical availability of participants, future studies may consider longer or more frequent interviews for more in-depth data collection. Finally, among the 10 conditions within the risk assessment, each individual condition may have had its own intrinsic utility and usability attributes. As individual conditions were not studied in isolation, the formative evaluation is not able to detect these potential differences.

This study describes the impact and process of implementing a novel family health history and CDS tool into the primary care, cancer survivorship and cancer genetics clinics. We found high acceptance and usability among clinicians, identified technical and clinical enhancements for improved usability and utility and identified topics for future research, including further study of the most effective mechanisms by which to deliver CDS to clinicians and the impact of CDS on the quality and content of the patient-clinician interaction. Future research will also include the evaluation of the patient experience and patient outcomes after use of *MyFamily*. Through continued development and evaluation of *MyFamily*, our multidisciplinary team will continue to seek maximally effective ways to deliver CDS on family health history to clinicians at the point of care through increasingly EMR-integrated information technology solutions. 

## 5. Conclusions

This formative evaluation sought to assess clinician usability, including the impact on clinic flow and perceptions of usefulness and value, and identify barriers and facilitators to maximizing clinical adoption to feed iterative improvement cycles. We conclude that primary care, cancer survivorship and cancer genetics clinicians found the *MyFamily* application usable, fitting naturally into their existing workflows and personal practice patterns, as well as of value, improving the quality and consistency of care provided. Barriers included a tension between length and detail, as well as practice-area specific challenges, e.g., the lack of consolidated cancer pedigrees for cancer genetic counselors. The model of iterative implementation and improvement cycles demonstrated here will be applied in future institutional efforts to integrate clinical genomic applications into practice and may be useful for other institutions considering system-wide implementation of family health history and genomic tools for personalizing medical management as part of the routine clinical armamentarium.

## Appendix A: Screen Shots of *MyFamily* Application

**Patient Interface: Disease Specific Question**

**Clinician Interface: Risk Justification and Clinical Considerations**

## Appendix B: Interview Guide

**Ease of Use**

1. One a scale of 1 to 5, with 1 being very easy and 5 being very difficult, how easy is it to use the Risk Reference (RR) in the patient encounter?
2. Are there parts of the RR that are easier or harder to use?
  - Which parts?
  - Why?
3. One a scale of 1 (very easy) to 5 (very difficult), how easy or hard is it to access the RR in the EMR?
4. One a scale of 1 (very easy) to 5 (very difficult), how easy or hard is it to approve the RR in the EMR?
5. One a scale of 1 (very easy) to 5 (very difficult), how easy or hard is it to understand the information in the RR?
6. Are there aspects of the RR that are difficult to understand?
  - Which parts?
  - How could these parts be improved?

**Perceived Usefulness and Value**

7. On a scale of 1 (very useful) to 5 (not very useful at all), how useful are *MyFamily* and the RR for your practice?
  - Which aspects of the RR are most useful for your practice?
  - Which aspects of the RR are least useful for your practice?
8. How useful will *MyFamily* be to other providers?
9. Is the length of the RR and amount of information okay, too little, or too long? Why or why not?
10. Is the level of difficulty of the information okay, too simple, or too complex?
  - (if too simple or too complex) Which parts are too (simple/complex)
  - How could this be improved?
11. Think about the clinical recommendations and risk assessment in the Risk Reference. On a scale of 1 to 5, with 1 being strongly agree and 5 being strongly disagree, how strongly do you agree with the clinical risk assessment and recommendations?
  - (if disagree) Please tell me more about your concerns.
  - Do you need additional information about how the recommendations were generated?
12. Do you think the RR increases or decreases quality of care?
13. On a scale of 1 (very helpful) to 5 (not very helpful at all), how helpful is the RR in meeting patient health maintenance requirements?
14. On a scale of 1 (very helpful) to 5 (not very helpful at all), how helpful is the RR in identifying patients appropriate for referral to subspecialties and other services?
15. Does the RR give you the information you need to appropriately identify and communicate with patients about a genetic risk?
  - Does the RR include enough additional or supportive educational content (e.g., why the patient is at increased risk, why the recommendation is considered best practice)
16. Does *MyFamily* and the RR change your confidence in identifying, managing, or communicating about genetic risks? Please describe your answer.

**Perceptions of Patient Value**

17. What have your patients told you about the process or experience of using *MyFamily* before the appointment?
18. Are there some kinds of patients who have an easier or harder time using *MyFamily*? If so, please describe.
19. Do you think *MyFamily* benefits CCF patients equally, or are there some patient populations who benefit more or less?
20. Do you recommend any changes to the patient interface of *MyFamily* for improved patient access and usability?

**Impact on Clinic Flow**

21. Approximately how many patients have you seen that have completed the *MyFamily* web questionnaire and have a RR available in the clinical encounter?
22. Can you please briefly describe how you have been using the RR in your clinic?
23. How has your clinical encounter changed since using RR?
24. Does the RR align with your current work flow?
  - If not, how did you change your work flow since the introduction of *MyFamily*?
  - What could be changed in the system to improve work flow using *MyFamily*?
25. Do you think using the RR saves time, increases time, or does not change your time spent in the clinical encounter?
26. Do you always use the RR if it is available? Why or why not?
27. What resources were needed to get ready for launch? Examples: Staff time, meetings, IT help/training.
  - Did you and your team have what they needed to launch? (Probe if needed: Is the level of *MyFamily* education and training that you received sufficient?)
28. What resources are needed to continue to use and maintain the program in the clinic?
29. What barriers or challenges has the team experienced in the implementation and use of *MyFamily*?
30. What facilitators have supported the successful implementation of *MyFamily*?
31. What is the impact of *MyFamily* on the clinical support staff (e.g., Medical Assistant)?

**Overall Impression**

32. What is your overall impression of *MyFamily* and the RR?
33. What is your favorite part of the program?
34. What is your least favorite part of the program?
35. Are there features of *MyFamily* or the RR that should be changed or added for improved usability and patient care? Please describe.
36. Do you have any concerns about using My Family and the RR?
37. What conditions should the *MyFamily* staff focus on next?
38. Any other comments?
